# Long non-coding RNA *Loc105611671* promotes the proliferation of ovarian granulosa cells and steroid hormone production upregulation of *CDC42*

**DOI:** 10.3389/fvets.2024.1366759

**Published:** 2024-03-04

**Authors:** Jinglei Wang, Hanying Chen, Yongsheng Zhang, Hong Shen, Xiancun Zeng

**Affiliations:** ^1^College of Animal Science and Technology, Shihezi University, Shihezi, Xinjiang, China; ^2^School of Pharmacy, Shihezi University, Shihezi, Xinjiang, China

**Keywords:** sheep, ovaries, *Loc105611671*, RNA-RNA interaction, *CDC42*

## Abstract

Granulosa cells (GCs) are essential for follicular development, and long non-coding RNAs (LncRNAs) are known to support the maintenance of this process and hormone synthesis in mammals. Nevertheless, the regulatory roles of these lncRNAs within sheep follicular GCs remain largely unexplored. This study delved into the influence of a *Loc105611671*, on the proliferation and steroid hormone synthesis of sheep ovarian GCs and the associated target genes *in vitro*. Cell Counting Kit-8 (CCK-8) gain-of-function experiments indicated that overexpression of *Loc105611671* significantly boosted GCs proliferation, along with estrogen (E_2_) and progesterone (P_4_) levels. Further mechanistic scrutiny revealed that *Loc105611671* is primarily localized within the cytoplasm of ovarian granulosa cells and engages in molecular interplay with *CDC42*. This interaction results in the upregulation of *CDC42* protein expression. Moreover, it was discerned that increased *CDC42* levels contribute to augmented proliferation of follicular granulosa cells and the secretion of E_2_ and P_4_. Experiments involving co-transfection elucidated that the concurrent overexpression of *CDC42* and *Loc105611671* acted synergistically to potentiate these effects. These findings provide insights into the molecular underpinnings of fecundity in ovine species and may inform future strategies for enhancing reproductive outcomes.

## 1 Introduction

GCs proliferation and growth are pivotal to the process of follicular development (1). This dynamic sequence begins with oocyte growth, followed by the recruitment of GCs (2, 3). As they progress toward ovulation, GCs undergo morphological changes and proliferate (4). As the predominant cell type within the follicle, GCs not only regulate their own proliferation but also contribute to follicle development by synthesizing hormones (E_2_, P_4_) (5, 6) and growth factors (7, 8). The process begins with the transformation of cholesterol, during which the *STAR* protein plays a pivotal role in translocating cholesterol to the inner mitochondrial membrane. At this juncture, the enzyme *P450scc* (*Cyp19a1* gene product) metabolizes cholesterol into pregnenolone (9). E_2_ production within GCs is facilitated by the enzymatic actions of *P450arom* aromatase and *17β-HSD* (10). With the development and maturation of the follicle, E_2_ levels rise, consequently stimulating GCs proliferation and differentiation. This not only impacts the quality and maturation of the oocyte but also plays a role in follicular evolution (11, 12). Furthermore, E_2_ facilitates the production of P_4_ by activating the P4 receptor, thus influencing GC proliferation and follicular development (13). The concentration of P_4_ fluctuates throughout the stages of follicular development, peaking during the maturation phase (14).

LncRNAs are a diverse class of RNAs over 200 bp in length, including intronic, intergenic, and antisense variants (15). These lncRNAs are known to play essential regulatory roles in mammalian reproduction, participating in cell proliferation (16), apoptosis (17), follicle development (18), oocyte maturation (19), and steroid hormone synthesis (20), underscoring their significance in reproductive biology. Recent studies indicate that lncRNAs contribute to reproductive processes by interacting with proteins and other RNAs. For example, *lncRNA PVT1* induces GC apoptosis by upregulating *Foxo3a* levels (21), while *lncRNA RP11-552 M11.4* collaborates with *BRCA2* to stimulate GC proliferation and prevent apoptosis (22). Despite these findings, research on mammalian reproduction has largely cantered on the discovery of novel lncRNAs (23–26), with limited investigation into the specific functions and mechanisms of lncRNAs, particularly in sheep ovarian GCs.

Our prior study revealed differential expression of *Loc105611671* in Qira black sheep during the pre-estrus and estrus phases (27), suggesting it may influence sheep reproductive capacities by regulating GC functions. Yet, the exact role of *Loc105611671* in sheep follicular GC regulation is still to be elucidated. To this end, we probed the impact of *Loc105611671* on GC proliferation and steroid hormone secretion by developing an *in vitro* cultured follicular GC model. Our study is designed to elucidate the complex regulatory mechanisms lncRNAs exert on sheep follicular development. Concurrently, it may lay the groundwork for identifying novel therapeutic approaches to reproductive disorders such as polycystic ovarian syndrome (PCOS), a condition that can result in ovulatory failure.

## 2 Materials and methods

### 2.1 Isolation and culture of GC

During the peak breeding season (August to October), healthy sheep ovaries from animals aged 1 to 1.5 years were sourced from a local abattoir in Shihezi, Xinjiang Uygur Autonomous Region, China. Mature dominant follicles were carefully selected, their follicular fluid aspirated and collected into Petri dishes containing (Dulbecco’s modified Eagle’s medium/nutrient mixture F-12 [DMEM/F12] (Gibco, France) medium. Oocytes were meticulously picked using a mouth pipette. The GCs were then transferred to erythrocyte lysis buffer to eliminate any red blood cells. The pelleted cells were washed twice with DMEM/F12 medium, cultured into Petri dishes enriched with 10% fetal bovine serum [FBS] (Gibco, France), 100 IU/mL penicillin, and 100 μg/mL streptomycin) aseptic culture at 37°C in a 5% CO_2_ atmosphere. After 48 h, non-adherent cells were removed by gentle medium replacement.

### 2.2 Quantitative reverse transcription-polymerase reaction (qRT-PCR)

Total RNA was extracted from cells using the TRIzol (Invitrogen, USA) assay. After the RNA samples were reverse transcribed into cDNA using the TransScript® First-Strand cDNA Synthesis SuperMix kit (Transgen, China) for quality assessment, they were assayed for gene expression by using the Perfectstart Green qPCR SuperMix PCR kit (Transgen, China) according to the user’s manual and a Roche Light Cycler 480 (Roche, Switzerland) to detect gene expression. The housekeeping gene, *GAPDH*, was used as an internal reference. Data were analyzed using the 2^-ΔΔCt^ method. All primers used in this study are listed in Supplementary Table S1.

### 2.3 Plasmid construction and transfection of GCs

GCs were cultured to 60–70% confluence and then transfected, or co-transfected, with Lipofectamine 2000 (Invitrogen, USA) for 72 h. The overexpression construct for *Loc105611671* was synthesized by cloning the full-length sequence into the EcoRI/BamHI sites of the pCDNA3.1-EGFP vector (denoted as LV-Loc105611671). Similarly, the *CDC42* overexpression plasmid was produced by inserting the CDC42 coding sequence into the same sites of the pCDNA3.1-EGFP vector (denoted as LV-CDC42). An empty pCDNA3.1-EGFP vector served as the control (denoted as LV-EGFP). The primers utilized for cloning are listed in Table 1.

**Table 1:** PCR Amplification primer information.

Gene	Primer sequence (5′-3′)
T7-Loc105611671 F	TAATACGACTCACTATAGGGAGAAGTGAGAGGAAGGCG
T7-Loc105611671 R	GATTATGATCTAGAGTCGCGG
T7-Loc105611671-AF	TAATACGACTCACTATAGGGGATTATGATCTAGAGTCGCGG
T7-Loc105611671-AR	AGAAGTGAGAGGAAGGCG
s-CDC42-F	aagctgtgaccggcgcctacgaattcGCCACCatgcagacaattaag
s-CDC42-R	CCccATCGATggACCGGTcgGGATCCtagcagcacacacctgcggc

### 2.4 RNA-protein pull-down assay

To synthesize biotinylated transcripts, we used the T7 *in vitro* transcription kit mMESSAGE mMACHINE® Kit (cat. AM1344, Invitrogen, USA) for transcription; the amplification primers are listed in Table 1. After purification using the RNeasy Mini Kit (cat. 74,104, QIAGEN, Germany), biotinylated RNA was added to the cell lysates. After treatment with RNase-free DNase I, the biotin-labeled *Loc105611671* was denatured for 3 min at 95°C, incubated on ice for 1 min, and rested at room temperature for 30 min to restore the secondary structure of the RNA. The RNA was then incubated with Streptavidin Magnetic Beads (cat. 21,344, Thermo, USA) for 1 h at room temperature and stirred in a clean test tube to form a magnetic bead-RNA mixture. Next, the extracted total GCs protein was added to the magnetic bead-RNA mixture and incubated for 1 h at room temperature with rotation to generate the magnetic bead-RNA-protein complexes. After washing and elution, RNA-bound proteins were collected and subsequently separated using SDS-PAGE elution and silver staining.

### 2.5 Cell proliferation analysis

For CCK-8 analysis (Transgen, China), post-transfection pellet cells were inoculated into 96-well plates (5 × 10^3^ cells per well). At 24, 48, and 72 h, 10 μL of CCK-8 reagent was added to each well and then cultured for 3 h. All experiments were performed in triplicate. The absorbance of each well was then measured at 450 nm using an enzyme marker (Thermo Fisher, USA).

### 2.6 Determination of steroid hormone concentrations by ELISA

At the indicated time points, post-transfection cell culture supernatants were collected using sheep enzyme E_2_ (sensitivity: less than 1.0 pg./mL, specificity: no cross-reactivity with other soluble structural analogs, reproducibility: intra-plate coefficient of variation less than 9%, inter-plate coefficient of variation less than 11%, cat. JL17550), P_4_ (sensitivity: less than 0.1 ng/mL, specificity: no cross-reactivity with other soluble structural analogs, repeatability: intra-plate coefficient of variation less than 9%, inter-plate coefficient of variation less than 11%, cat. JL22308) ELISA was determined by absorbance at 450 nm by an enzyme labeller (Thermo Fisher, USA). Sheep E_2_ ELISA kit and P_4_ ELISA kit were purchased from Jianglai Biotechnology Co Ltd. (Jianglai, China).

### 2.7 Subcellular localization

For nuclear and cytoplasmic RNA isolation, the nuclear and cytoplasmic fractions were collected and extracted using the nuclear/cytoplasmic separation kit (cat. HR0241, Biolab, China) according to the manufacturer’s instructions. The expression of *Loc105611671* and *CDC42* in cytoplasmic and nuclear RNA of GCs was measured using qRT-PCR as mentioned above. *XIST* and *ACTB* were used as positive controls for the nucleus and cytoplasm, respectively.

### 2.8 Western blot analysis

Total proteins were extracted from the cells using RIPA lysis buffer (containing 1% PMSF) and then denatured by heating at 100°C. Equal amounts of protein were separated by SDS-PAGE and then transferred to PVDF membranes. After blocking with 5% skimmed milk and incubation with primary and secondary antibodies, immunoreactive bands on the membrane were reacted with ECL solution and detected by chemiluminescence imaging using a Tennant chemiluminescence imager (Tanon, China). The primary antibodies used in this study were anti-GAPDH (1:5000, cat. GTX100118, GeneTex, USA), anti-CDC42 (1:1000, cat. GTX134588, GeneTex, USA) and goat an-ti-rabbit (1:10000, cat. YK2231, Y&K Bio, China).

### 2.9 Dual luciferase reporter gene assay

293 T cells were cultured in 96-well plates (5 × 10^3^ cells per well). When the cell density reached 50–70%, the transfection plasmids were cotransfected individually into the cells. After 48 h of transfection, luciferase activity was measured using a dual luciferase reporter gene assay system (BioTek, USA).

### 2.10 Statistical analysis

All experimental data were analyzed using GraphPad Prism 8.0 software (GraphPad Inc) or SPSS 26.0 system (SPSS Inc). An independent samples t-test was used to analyze the statistical differences between the two groups. Each experiment was repeated three times. *p*-values less than 0.05 were considered statistically significant as follows: “*” *p* < 0.05, “**” *p* < 0.01, and “***” *p* < 0.001.

## 3 Results

### 3.1 Overexpression of *Loc105611671* enhances GCs proliferation

**Figure 1:**
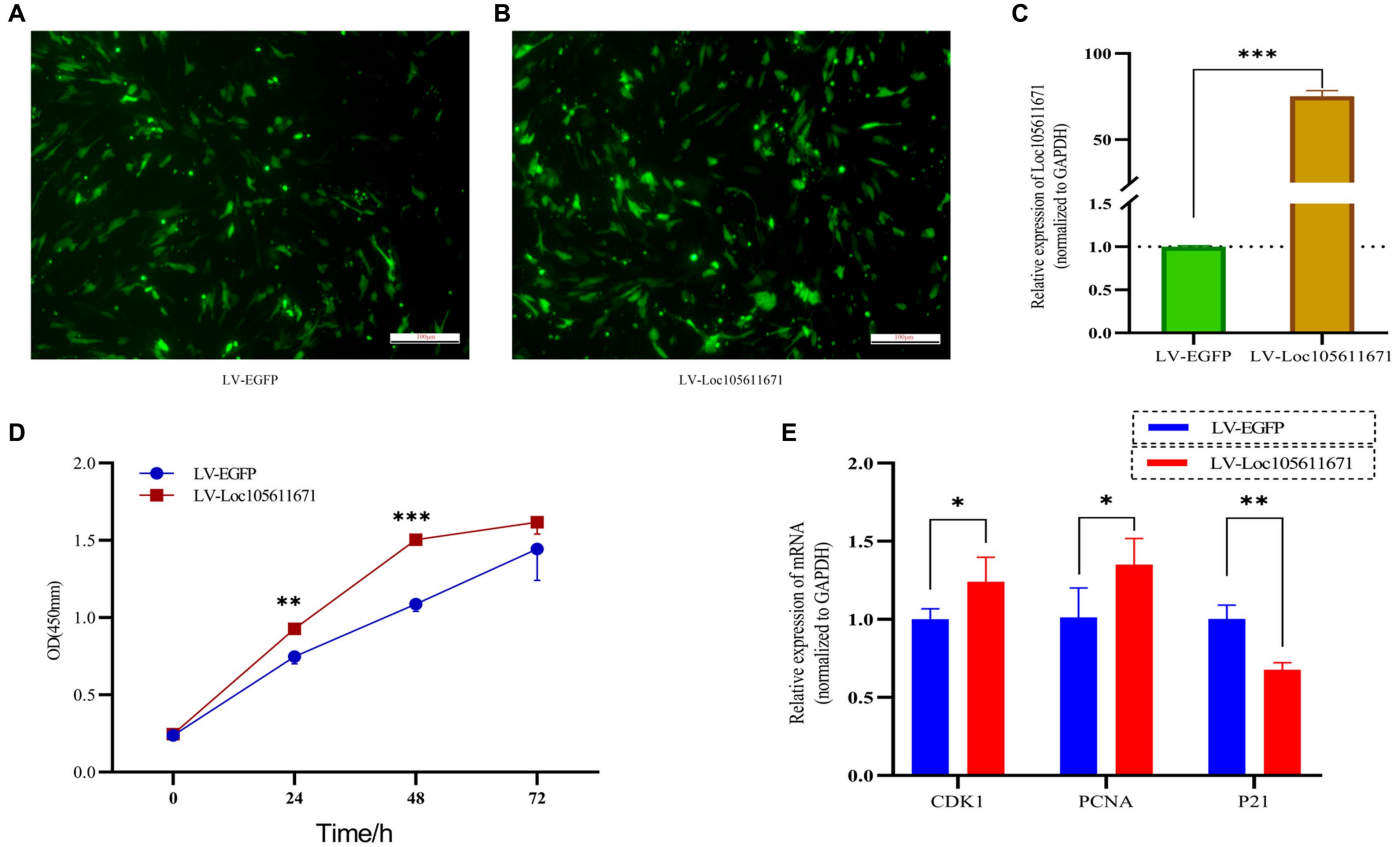
Overexpression of *Loc10561167* enhances GCs proliferation. **(A–C)** The qRT-PCR method was utilized to assess *Loc105611671* expression levels in GCs following transfection with LV-Loc105611671 vs. LV-EGFP (*n* = 4). **(D)** CCK-8 was employed to assess the growth level resulting from *Loc105611671* overexpression in GCs (*n* = 3). **(E)** Transfection of GCs with LV-Loc105611671 was carried out for 24 h, after which mRNA levels of *CDK1*, *PCNA*, and *P21* were measured (*n* = 4). Data represent the mean ± SD. *p < 0.05, **p < 0.01, ***p < 0.001.

To elucidate the regulatory effects of *Loc105611671* on GCs *in vitro*, we used overexpression plasmids to overexpress *Loc105611671*. The proliferation rate of GCs was analyzed using CCK-8 assay. The efficiency of *Loc105611671* overexpression based on the pCDNA3.1-EGFP vector was confirmed using qRT-PCR (Figures 1A–C). The results demonstrated that *Loc105611671* upregulation significantly enhanced GC proliferation at 24 h (0.747 ± 0.046 vs. 0.928 ± 0.026, *p* < 0.01) and at 48 h (1.088 ± 0.047 vs. 1.504 ± 0.032, *p* < 0.001) compared with the control. After 72 h (1.445 ± 0.204 vs. 1.617 ± 0.077, *p* > 0.05), the proliferation rates between the test and control groups began to align (Figure 1D). Additionally, the overexpression of *Loc105611671* notably upregulated the mRNA expression levels of *CDK1* and *PCNA*, both positive cell cycle regulators, while downregulating *P21*, a cell cycle inhibitor (Figure 1E). These results indicate that *Loc105611671* plays a pivotal role in the proliferation of GCs.

### 3.2 *Loc105611671* promotes E2 and P4 synthesis in GCs

**Figure 2:**
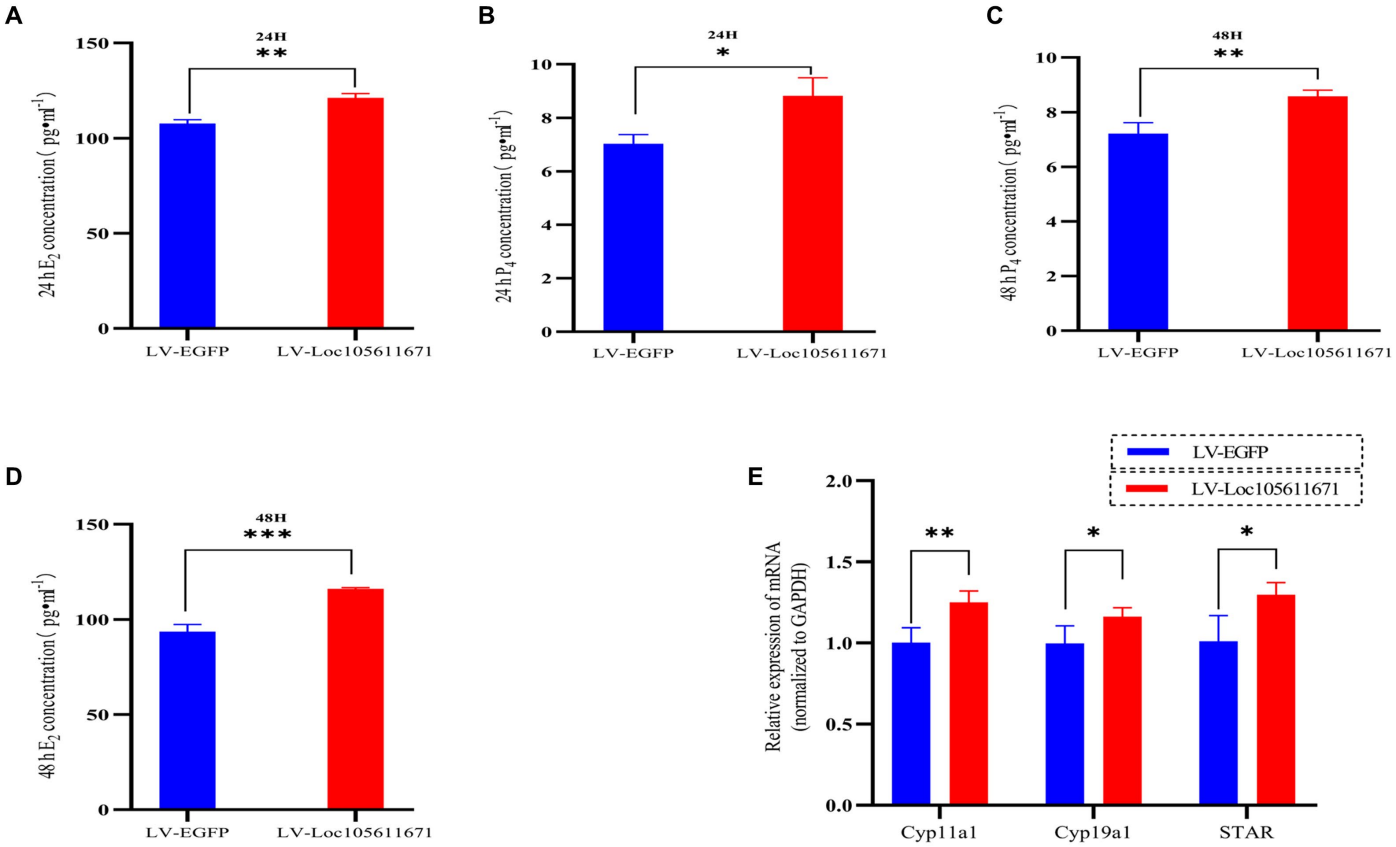
*Loc105611671* regulates steroid secretion through GCs. The 24 h **(A,C)** and 48 h **(B,D)** E2 and P_4_ concentrations in cell supernatants were detected using ELISA (*n* = 3); **(E)** qRT-PCR was used to detect the expression levels of the genes important for steroid hormone synthesis, *STAR*, *Cyp11a1*, and *Cyp19a1* mRNA expression (*n* = 4). Data represent the mean ± SD. *p < 0.05, **p < 0.01, or ***p < 0.001.

To determine the effect of *Loc105611671* expression on steroid hormone production, we performed ELISA to measure the E_2_ and P_4_ levels in ovarian GCs 24 h and 48 h after transfection with LV-Loc105611671. A significant elevation in E_2_ secretion was detected at 24 h (107.695 ± 2.022 vs. 121.170 ± 2.242 pg./mL, *p* < 0.01) and 48 h (93.653 ± 3.718 vs. 116.064 ± 0.638 pg./mL, *p* < 0.001) in the LV-Loc105611671 group compared to controls (Figures 2A,B). Similarly, P_4_ secretion levels were significantly increased following *Loc105611671* overexpression at 24 h (7.032 ± 0.344 vs. 8.822 ± 0.678 ng/mL, *p* < 0.05) and at 48 h (7.211 ± 0.401 vs. 8.581 ± 0.228 ng/mL, *p* < 0.01) (Figures 2C,D). Moreover, a marked upregulation was observed in the mRNA expression of *STAR*, *Cyp11a1*, and *Cyp19a1*, genes involved in steroidogenesis (Figure 2E). These data suggest that *Loc105611671* plays an important role in steroid hormone synthesis.

### 3.3 RNA pull-down assay to identify *Loc105611671* binding proteins

**Figure 3:**
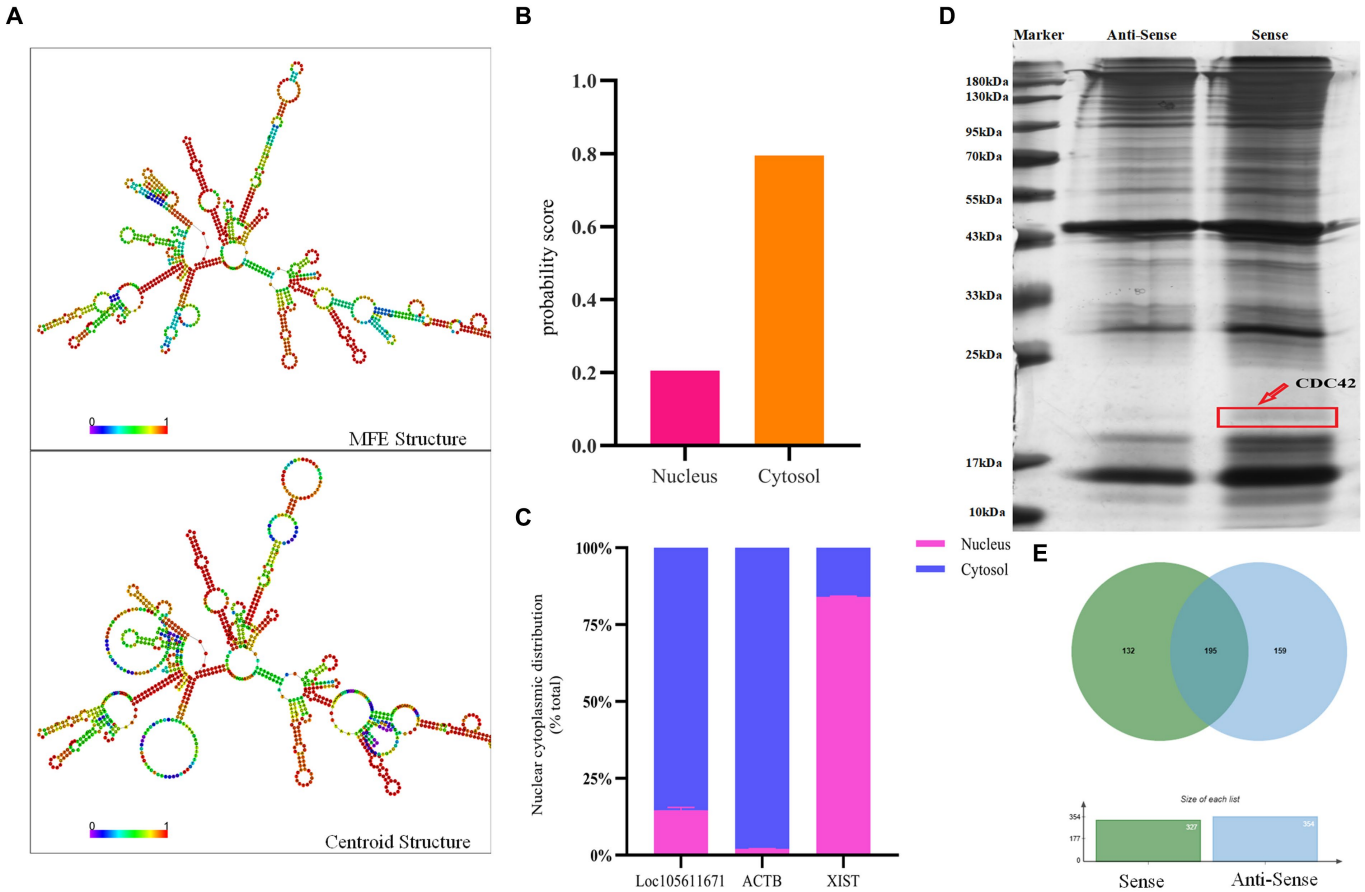
RNA pull-down captures *Loc105611671* binding protein. **(A)** Secondary structure of *Loc105611671* transcripts in MFE and Centroid modes predicted by the online tool RNAfold. **(B)** *Loc105611671* subcellular localization in granule cells predicted by the online tool iLoc-LncRNA. **(C)** Subcellular localization of *Loc105611671* (*n* = 3). **(D)** Silver-stained SDS-PAGE gels for proteins extracted from biotin-labeled *Loc105611671* and antisense RNA, and two lanes for mass spectrometry. **(E)** Venn diagram of the results of mass spectrometry analysis.

Long non-coding RNAs (lncRNAs) enact their biological roles through interactions with diverse biomolecules, and these interactions are often influenced by the RNA’s subcellular localization. According to the iLoc-LncRNA database,^1^ *Loc105611671* is predominantly found in the cytoplasm, a finding further substantiated by nucleoplasmic separation experiments (Figures 3B,C). To explore the secondary structure of *Loc105611671*, we utilized the RNAfold tool,^2^ which predicted Y-shaped structures in both minimally free energy (MFE) and centroid modes (Figure 3A). The cytoplasmic localization of *Loc105611671*, suggestive of its potential for protein interaction, prompted us to perform an RNA pull-down assay followed by mass spectrometry (Figure 3D), which identified 132 putative binding proteins (PBPs) (Figure 3E; Supplementary Table S2).

**Figure 4:**
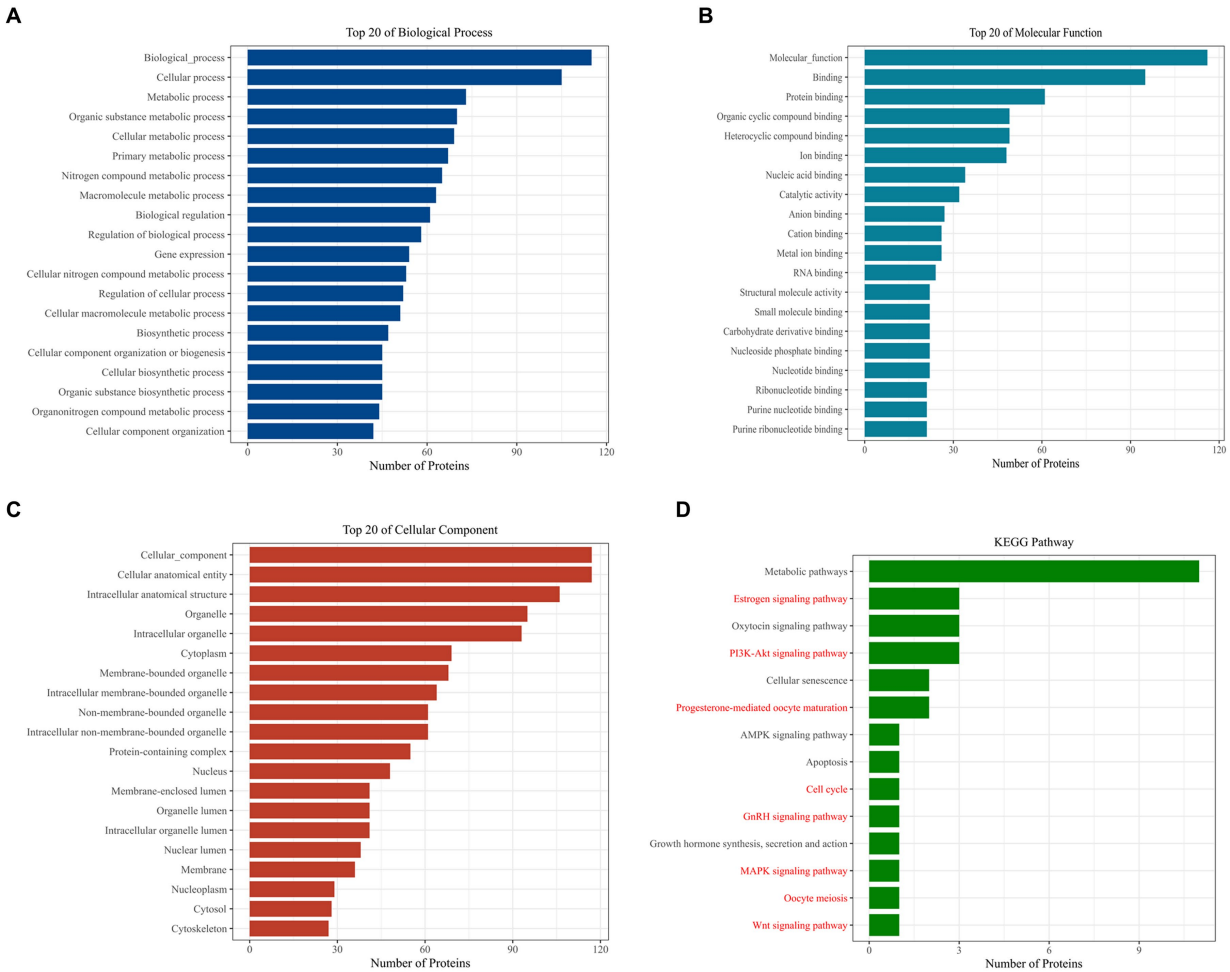
Bioinformatics analysis to identify proteins. GO analysis of *Loc105611671* potential binding proteins **(A–C)**. KEGG analysis of *Loc105611671* potential binding proteins **(D)**, The red pathway is more common in mammalian reproduction studies.

Further analysis of *Loc105611671* PBPs using Gene Ontology (GO) and Kyoto Encyclopedia of Genes and Genomes (KEGG) revealed enrichment in biological processes (BPs) such as metabolism, biological regulation, process regulation, and gene expression (Figure 4A). Molecular functions (MF) were predominantly associated with protein binding, nucleic acid binding, catalytic activity, RNA binding, and nucleotide binding (Figure 4B). Cellular components (CC) included intracellular organelles, the cytoplasm, and protein-containing complexes (Figure 4C). KEGG enrichment analysis identified 14 signaling pathways potentially linked to GC proliferation and hormone production, including PI3K-Akt signaling pathway, Wnt signaling pathway, GnRH signaling pathway, MAPK signaling pathway, oocyte meiosis and Cell cycle, etc. (Figure 4D).

### 3.4 Direct interaction between *Loc105611671* and *CDC42* protein with positive regulatory effects

**Figure 5:**
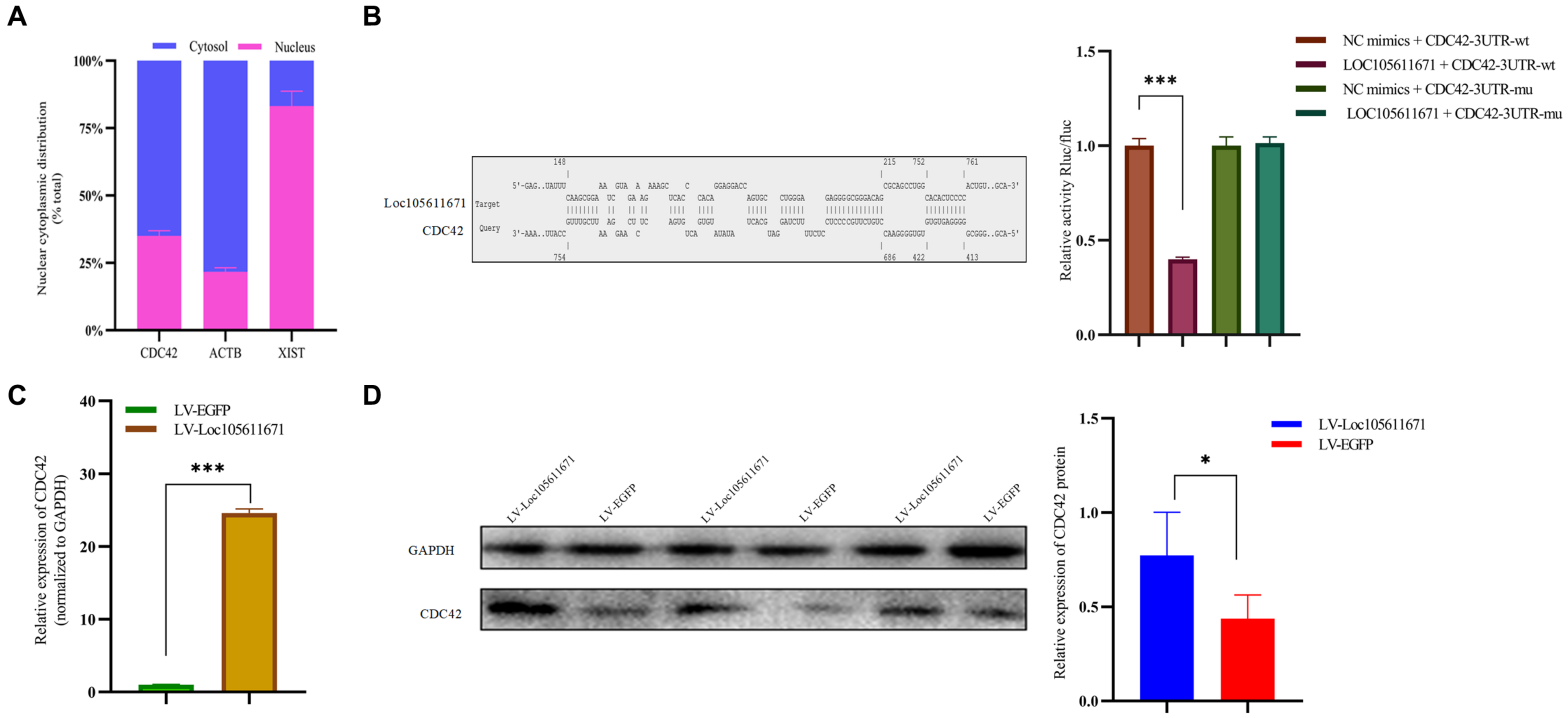
*Loc105611671* interacts with *CDC42* and positively regulates its protein and mRNA expression levels. **(A)** Subcellular localization of *CDC42* was determined (*n* = 3). **(B)** The binding of *Loc105611671* to the 3’UTR of *CDC42* mRNA was demonstrated using the online IntaRNA tool and dual luciferase reproter gene assay (*n* = 3). **(C,D)** following *Loc105611671* overexpression, significant increase in protein and mRNA expression levels of *CDC42* was observed in GCs (*n* = 3). Data represent the mean ± SD. *p < 0.05, ***p < 0.001.

KEGG analysis identified six proteins as candidate binding proteins, of which *CDC42* was involved in signaling pathways significant in cell proliferation and hormone production (Supplementary Table S3). Subsequent functional queries of these RNA-binding proteins revealed that *CDC42* participates in the regulation of cell proliferation, differentiation, and apoptosis. Therefore, *CDC42*, which showed the highest reliability, was selected for subsequent studies. Subsequently, we used the online tool IntaRNA^3^ to predict a possible interaction between *Loc105611671* and *CDC42* 3’UTR. This interaction was confirmed using a dual-luciferase reporter gene assay (Figure 5B). Nucleocytoplasmic separation of *CDC42* demonstrated its presence in both the cytoplasm and nucleus, predominantly in the cytoplasm, providing additional support for the intracellular interaction with *Loc105611671* (Figure 5A).

To investigate the promotion of GC proliferation and steroid hormone production through interaction with *CDC42*, we examined whether *Loc105611671* would affect the expression of *CDC42*. Our findings revealed that overexpression of *Loc105611671* significantly upregulated *CDC42* protein and mRNA levels in GCs (Figures 5C,D). These outcomes indicate that *Loc105611671* directly interacts with *CDC42*, augmenting its expression, and suggesting that *CDC42* is a downstream effector of *Loc105611671*.

### 3.5 Manipulation of CDC42 expression regulates GCs proliferation

**Figure 6:**
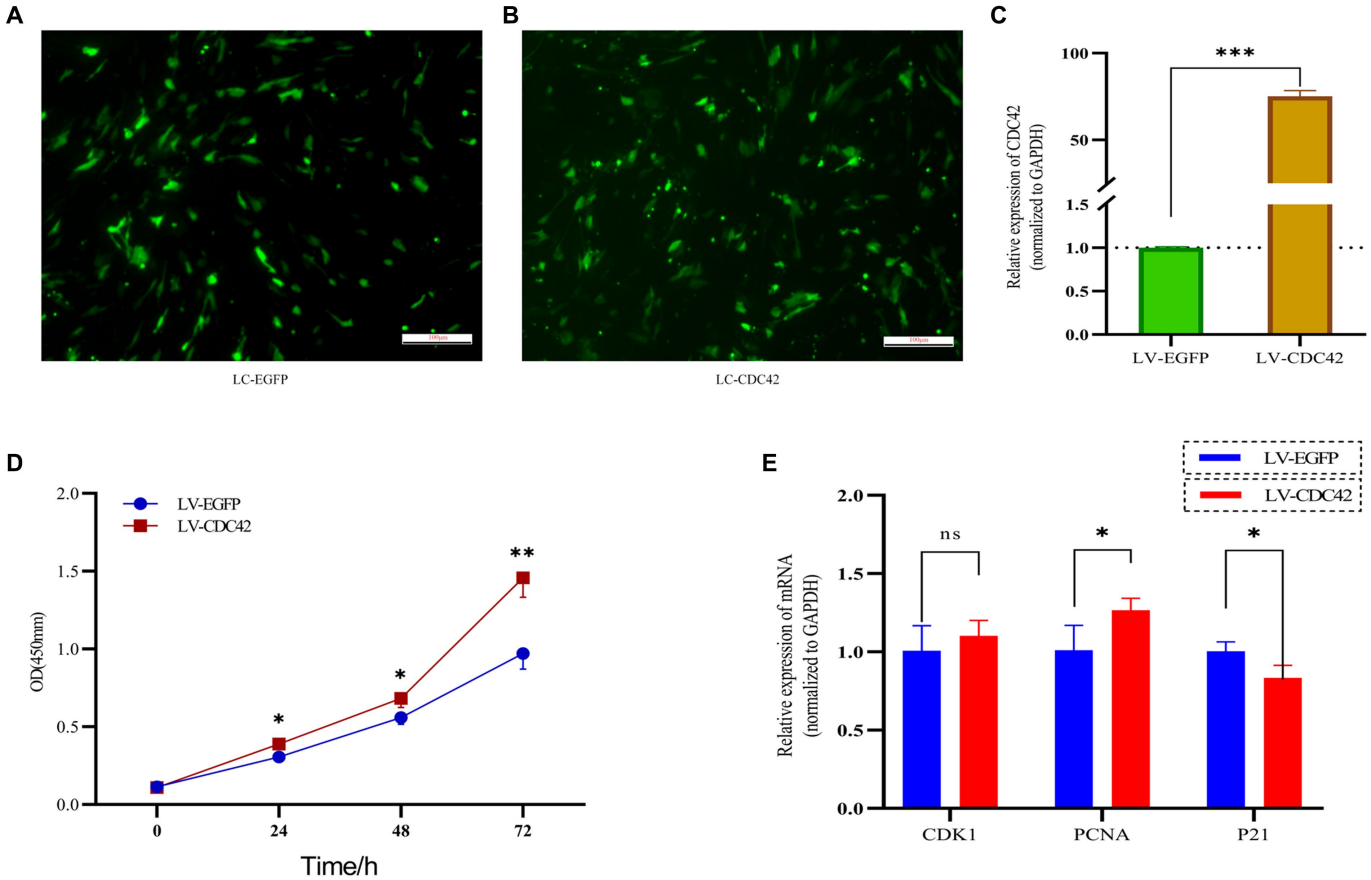
Overexpression of *CDC42* promotes GCs proliferation. **(A–C)** Overexpression of *CDC42* transfection efficiency, qRT-PCR to detect the relative expression level of *CDC42* in GCs, LV-CDC42 vs. LV-EGFP (*n* = 4). **(D)** Growth levels of *CDC42* overexpressing GCs were detected by CCK-8 (*n* = 3). **(E)** GCs were transfected with LV-CDC42 for 24 h and mRNA levels of *CDK1*, *PCNA*, and *P21* were determined (*n* = 4). Data represent the mean ± SD. ^ns^*p* > 0.05, *p < 0.05, **p < 0.01, ***p < 0.001.

*CDC42* is highly expressed in the oocytes of activated follicles, and its overexpression promotes the growth of primordial follicles in mouse ovaries (28). We hypothesized that *CDC42* would act as a crucial downstream regulator of *Loc105611671* and play a role in the promotion of GC proliferation. To verify this hypothesis, we elevated *CDC42* expression levels in GCs (Figures 6A–C). CCK-8 results showed that GCs proliferated at a faster rate after transfection with *CDC42* than the controls did at 24 h (0.306 ± 0.038 vs. 0.388 ± 0.008, *p* < 0.05), (0.559 ± 0.044 vs. 0.683 ± 0.059, *p* < 0.05) 48 h, and 72 h (0.971 ± 0.101 vs. 1.458 ± 0.126, *p* < 0.01) (Figure 6D). In addition, *CDC42* overexpression significantly increased the mRNA level of *PCNA* but had no significant effect on *CDK1* expression, and the expression of the negative regulator of proliferation, *P21*, was significantly downregulated (Figure 6E). These results suggest that *Loc105611671* plays a crucial role in follicular development by promoting GC proliferation.

### 3.6 CDC42 is involved in the regulation of steroid hormone production in GCs

**Figure 7:**
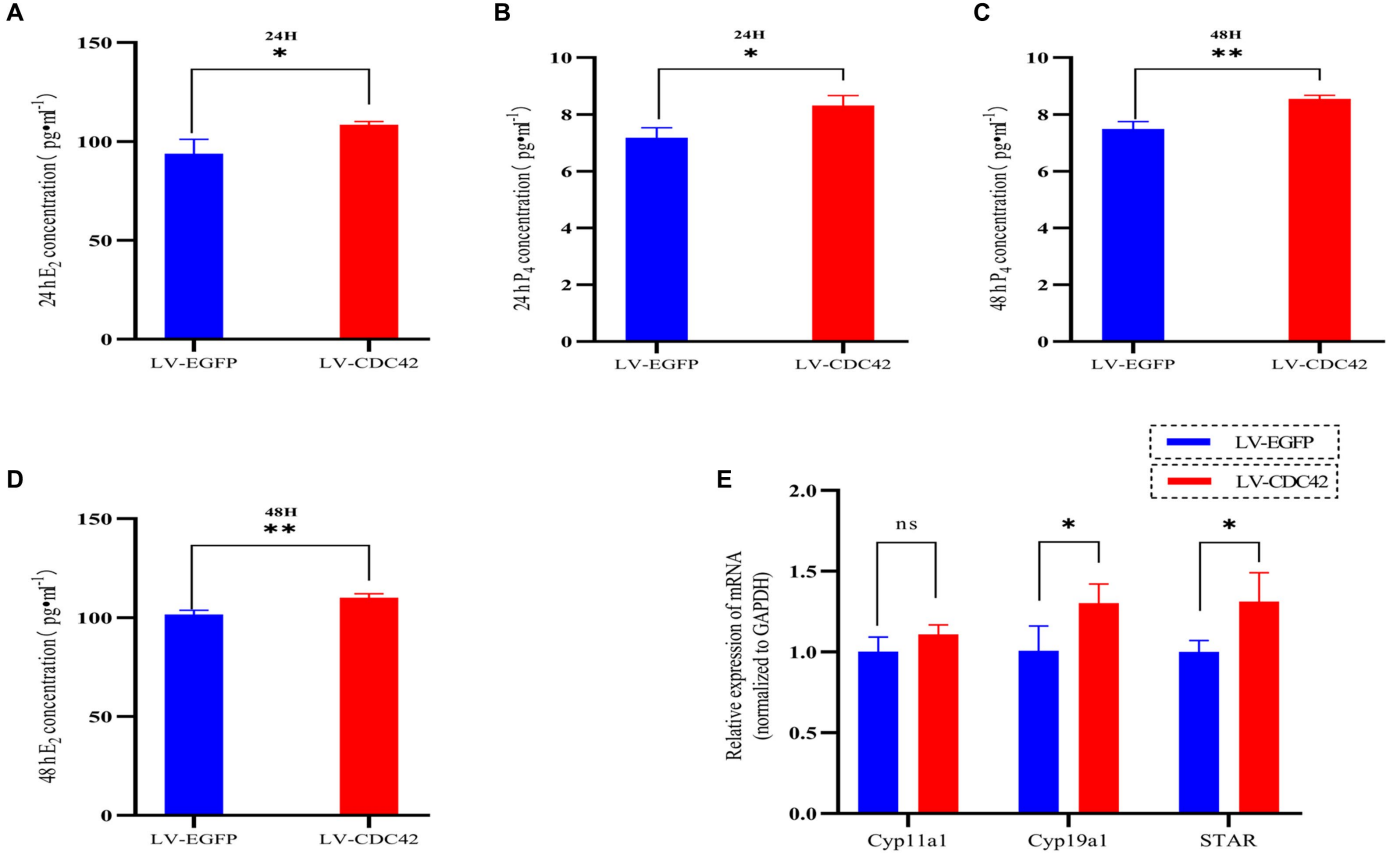
*CDC42* is implicated in regulating steroid hormone production in GCs. The concentrations of E2 and P_4_ in cell supernatants were detected via ELISA for 24 h **(A,C)** and 48 h **(B,D)** (*n* = 3). **(E)** Gene expression analysis of key steroid hormone synthesis genes was performed using qRT-PCR (*n* = 4). Data represent the mean ± SD. ^ns^*p* > 0.05, *p < 0.05, **p < 0.01.

We further investigated the effect of *CDC42* in GCs on E_2_ and P_4_ hormone production. The results showed that overexpression of *CDC42* significantly increased the level of E_2_ secretion at both 24 h (93.908 ± 7.179 vs. 108.513 ± 1.617 pg./mL, *p* < 0.05) and 48 h (101.583 ± 2.082 vs. 110.048 ± 2.013997 pg./mL, *p* < 0.01) (Figures 7A,B). Similarly, P_4_ secretion was significantly increased at both 24 h (7.183 ± 0.345 vs. 8.312 ± 0.352 ng/mL, *p* < 0.05) and 48 h (7.489 ± 0.258 vs. 8.543 ± 0.128 ng/mL, *p* < 0.01) (Figures 7C,D). Furthermore, the elevated *CDC42* expression markedly increased *Cyp19a1* and *STAR* mRNA levels, without affecting *Cyp11a1* expression (Figure 7E). In conclusion, the gain-of-function experiments demonstrated that high expression of *CDC42* in transfected GCs could promote E_2_ and P_4_ hormone production.

### 3.7 *Loc105611671* promotes GCs proliferation and E2 and P4 hormone production via CDC42

**Figure 8:**
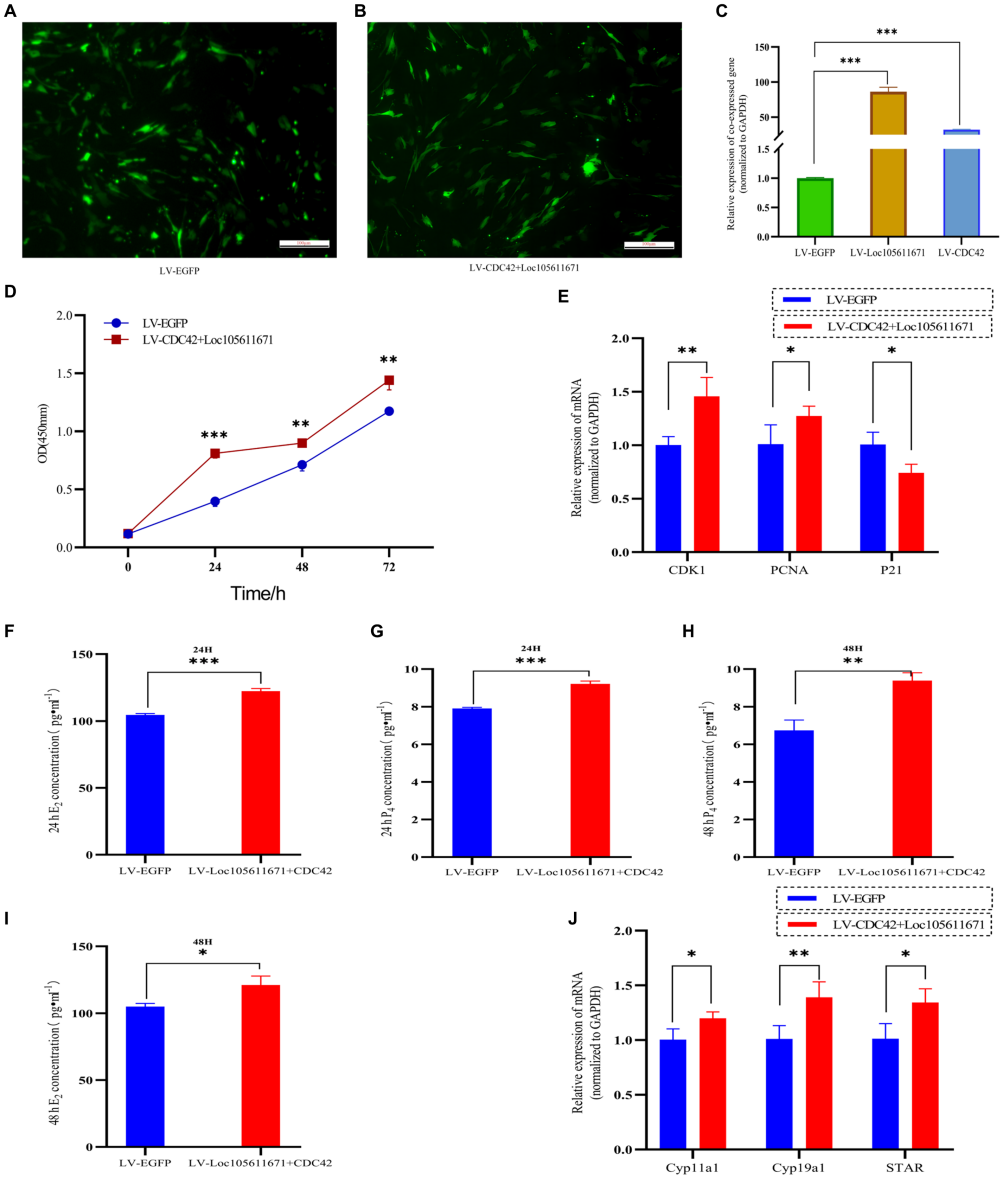
*Loc105611671* regulates GCs proliferation and E_2_ and P_4_ hormone production by promoting increased *CDC42*. **(A–C)** qRT-PCR was performed to detect the relative expression levels of co-transfected target plasmids in GCs (*n* = 4). **(D)** CCK-8 assay to detect the growth level of co-transfected LV-CDC42 + Loc105611671 granulocytes (*n* = 3). **(E)** Determination of mRNA levels of *CDK1*, *PCNA* and *P21* in co-transfected GCs for 24 h (*n* = 3). E_2_ and P_4_ concentrations (*n* = 3) in GCs after 24 h **(F,H)** 48 h **(G,I)** of co-transfection. **(J)** mRNA expression levels of *Cyp11a1*, *Cyp19a1*, and *STAR*, genes important for steroid hormone synthesis, were detected by qRT-PCR (*n* = 4). Data represent the mean ± SD. *p < 0.05, **p < 0.01, or ***p < 0.001.

Considering that *Loc105611671* directly to *CDC42* protein and has similar biological functions in regulating GCs proliferation and E_2_ and P_4_ hormone production. Therefore, it was hypothesized that *Loc105611671* plays a proliferative role in GCs by interacting with *CDC42*. To test this hypothesis, we performed co-transfection experiments (Figures 8A–C) and examined its biological functions. The results showed that simultaneous overexpression of *Loc105611671* and *CDC42* was observed at 24 h (0.281 ± 0.039 vs. 0.691 ± 0.042, *p* < 0.001), 48 h (0.596 ± 0.047 vs. 0.778 ± 0.034, *p* < 0.01) and 72 h (1.173 ± 0.039 vs. 1.440 ± 0.082, *p* < 0.01) significantly promoted granulocyte proliferation (Figure 8D). Meanwhile, E_2_ and P_4_ hormone production assays showed that overexpression of *Loc105611671* and *CDC42* significantly increased E_2_ secretion at 24 h (104.610 ± 0.996 vs. 122.285 ± 2.098 pg./mL, *p* < 0.001) and 48 h (104.916 ± 2.3697 vs. 121.101 ± 6.721 pg./mL, *p* < 0.05) (Figures 8F,G). Similarly, the level of P_4_ hormone production was increased at 24 h (7.908 ± 0.059 vs. 9.216 ± 0.144 ng/mL, *p* < 0.001) and 48 h (6.742 ± 0.546 vs. 9.388 ± 0.419 ng/mL, *p* < 0.01) (Figures 8H,I). In addition, mRNA levels of proliferation-related genes were significantly elevated after cotransfection, and the expression of genes related to the process of steroid hormone synthesis was also promoted (Figures 8E,J). Collectively, these data strongly suggest that *Loc105611671* regulates GC cell proliferation and E_2_ and P_4_ hormone production by targeting *CDC42*.

## 4 Discussion

In this investigation, our findings indicate that *Loc105611671* plays a crucial role in promoting the proliferation of sheep ovarian GCs and the biosynthesis of E_2_ and P_4_. *Loc105611671* enhances GC proliferation and hormone secretion by modulating *CDC42* expression and directly interacting with it.

GCs as the ovarian’s primary functional units, are instrumental in follicular growth and maturation, offering nutritional support and secreting hormones imperative for follicular development (5, 7, 29). Notably, *Loc105611671* was identified in the follicles of Qira Black sheep during estrus, implicating a regulatory effect on follicular development through GC modulation (27). Consequently, there is a compelling need for further research into *Loc105611671* roles in GC proliferation and steroid hormone production. The functionality and mechanistic action of *Loc105611671* in sheep GCs, however, have remained uncharted. Bridging this knowledge gap, we isolated GCs from ovine follicles to explore the impact of *Loc105611671* overexpression on their proliferation and steroidogenesis. Functional assays revealed that elevated *Loc105611671* levels not only stimulated GCs proliferation but also upregulated the cell cycle regulators *CDK1* and *PCNA* and downregulated *P21*. Intriguingly, these effects attenuated after 72 h, potentially due to the dilution of exogenous genes amidst ongoing GCs division. Correspondingly, *Loc105611671* overexpression significantly boosted E_2_ and P_4_ concentrations and the expression of *STAR*, *Cyp11a1*, and *Cyp19a1*, highlighting its role in promoting steroidogenesis. In conclusion, these findings suggest that *Loc105611671* is an important regulator of follicular development.

LncRNA function correlates with its subcellular localization (30). Nucleoplasmic separation experiments have demonstrated that *Loc105611671* is enriched in the cytoplasm of GCs, suggesting its protein-binding capacity. This regulatory network of LncRNAs has been identified in the literature on GCs’ functional mechanisms. For example, *lncRNA ZNF674-AS1* affects GC proliferation by interacting with *ALDOA* (31). *lnc-GULP1-2:1* was also found to bind directly to *COL3A1* and affect GC proliferation by regulating *COL3A1* expression and localization (32). In this study, we used RNA pull-down, mass spectrometry, and dual-luciferase gene assay experiments to further reveal that *CDC42* directly binds to and interacts with *Loc105611671* in GCs. To our knowledge, we are the first to report *CDC42* acting as an lncRNA-binding protein in sheep GCs. However, understanding the detailed protein-binding motifs in *Loc105611671* requires further characterization.

*CDC42* is a member of the Rho GTPase family and is involved in various cellular functions and signaling pathways, including cell proliferation (33), apoptosis (34), and the cell cycle (35). In particular, *CDC42* plays a crucial role in establishing mammalian oocyte polarity. For example, *CDC42* deficiency disrupts oocyte maturation during development *in vitro* (36). The subcellular localization of *CDC42* during primordial follicle activation is of interest. In dormant primordial follicles, *CDC42* is specifically expressed in oocyte cytoplasm. When primordial follicles were activated, *CDC42* expression on the oocyte membrane increased significantly (28). Furthermore, *CDC42* interacts with epidermal growth factor *EGF* to improve primordial follicle activation in mice by regulating the PI3K signaling pathway (37). In this study, elevated levels of *CDC42* were associated with enhanced cell proliferation, potentially contributing to improved GCs function. These findings are consistent with those in the literature on various animal species (33, 38, 39). As *CDC42* can induce steroid hormone activity, an interactive relationship is implied between *CDC42* expression and steroid hormone production (40, 41). Therefore, in this study, we investigated steroid hormone synthesis. The upregulation of *CDC42* resulted in a significant increase in E_2_ and P_4_ levels and *STAR* and *Cyp19a1* expression, indicating that *CDC42* promotes steroid hormone production in GCs. Subsequently, *In vivo* co-transfection experiments affirmed that *Loc105611671* and *CDC42* co-overexpression synergistically intensified GC proliferation and hormone secretion beyond their individual effects.

Unfortunately, although our findings have demonstrated that *Loc105611671* plays a positive regulatory role in follicular granulosa cell proliferation through *CDC42*, the specific molecular mechanisms behind this phenomenon remain unknown. *CDC42* is a crucial kinase within the MAPK signaling pathway and it influences a range of cellular behaviors. Therefore, our future research will concentrate on investigating how the *Loc105611671-CDC42*-regulated MAPK signaling pathway impacts follicular granulosa cells.

## 5 Conclusion

In conclusion, our study establishes that *Loc105611671* enhances follicular granulosa cell proliferation and steroidogenesis through its interaction with *CDC42*. This novel mechanism of lncRNA-protein interaction deepens our understanding of the physiological roles played by lncRNAs in coordinating GCs function and the complex follicular maturation process. Moreover, these findings lay the groundwork for the development of innovative therapeutic strategies targeting reproductive diseases.

## Data availability statement

The original contributions presented in the study are included in the article/Supplementary material, further inquiries can be directed to the corresponding author.

## Ethics statement

Ethical review and approval was not required for the study on animals in accordance with the local legislation and institutional requirements.

## Author contributions

JW: Conceptualization, Data curation, Software, Validation, Visualization, Writing – original draft, Writing – review & editing. HC: Investigation, Software, Supervision, Writing – original draft. YZ: Conceptualization, Investigation, Writing – review & editing. HS: Investigation, Methodology, Writing – review & editing. XZ: Conceptualization, Funding acquisition, Project administration, Resources, Supervision, Writing – review & editing.

## Footnotes

^1^ http://lin-group.cn/server/iLoc-LncRNA/home.php

^2^ http://rna.tbi.univie.ac.at/cgi-bin/RNAWebSuite/RNAfold.cgi

^3^ http://rna.informatik.uni-freiburg.de/IntaRNA/Input.jsp

## References

[1] Feng GH Liu J Lu ZT Li YK Deng M Liu GB. miR-450-5p and miR-202-5p synergistically regulate follicle development in black goat. Int J Mol Sci. (2023) 24:20. doi: 10.3390/ijms24010401, PMID: 36613843 PMC9820456

[2] Jolly PD Smith PR Heath DA Hudson NL Lun S Still LA. Morphological evidence of apoptosis and the prevalence of apoptotic versus mitotic cells in the Membrana granulosa of ovarian follicles during spontaneous and induced atresia in ewes. Biol Reprod. (1997) 56:837–46. doi: 10.1095/biolreprod56.4.837, PMID: 9096863

[3] Matsuda F Inoue N Manabe N Ohkura S. Follicular growth and atresia in mammalian ovaries: regulation by survival and death of granulosa cells. J Reprod Dev. (2012) 58:44–50. doi: 10.1262/jrd.2011-012, PMID: 22450284

[4] Lintern-Moore S Moore GP. The initiation of follicle and oocyte growth in the mouse ovary. Biol Reprod. (1979) 20:773–8. doi: 10.1095/biolreprod20.4.773 454765

[5] Chauvin S Cohen-Tannoudji J Guigon CJ. Estradiol signaling at the heart of Folliculogenesis: its potential deregulation in human ovarian pathologies. Int J Mol Sci. (2022) 23:20. doi: 10.3390/ijms23010512, PMID: 35008938 PMC8745567

[6] Chou CH Chen MJ. The effect of steroid hormones on ovarian follicle development In: Litwack G, editor. Ovarian cycle. Vitamins and hormones, vol. 107. San Diego: Elsevier Academic Press Inc. (2018). 155–75. 10.1016/bs.vh.2018.01.013 29544629

[7] Monte APO Santos JM Menezes VG Gouveia BB Lins T Barberino RS. Growth differentiation Factor-9 improves development, mitochondrial activity and meiotic resumption of sheep oocytes after *in vitro* culture of secondary follicles. Reprod Domest Anim. (2019) 54:1169–76. doi: 10.1111/rda.13485, PMID: 31173652

[8] Richani D Gilchrist RB. The epidermal growth factor network: role in oocyte growth, maturation and developmental competence. Hum Reprod Update. (2018) 24:1–14. doi: 10.1093/humupd/dmx029, PMID: 29029246

[9] Fuentes N Silveyra P. Estrogen receptor signaling mechanisms. Adv Protein Chem Struct Biol. (2019) 116:135–70. doi: 10.1016/bs.apcsb.2019.01.001 31036290 PMC6533072

[10] Simpson ER Clyne C Rubin G Boon WC Robertson K Britt K. Aromatase – a brief overview. Annu Rev Physiol. (2002) 64:93–127. doi: 10.1146/annurev.physiol.64.081601.142703, PMID: 11826265

[11] Engelhardt H Smith KB McNeilly AS Baird DT. Expression of messenger ribonucleic acid for inhibin subunits and ovarian secretion of inhibin and estradiol at various stages of the sheep estrous cycle. Biol Reprod. (1993) 49:281–94. doi: 10.1095/biolreprod49.2.281 8373951

[12] Pierre A Mayeur A Marie C Cluzet V Chauvin J Frydman N. Estradiol regulates mRNA levels of estrogen receptor Beta 4 and Beta 5 isoforms and modulates human granulosa cell apoptosis. Int J Mol Sci. (2021) 22:16. doi: 10.3390/ijms22095046, PMID: 34068748 PMC8126246

[13] Ting AY Xu J Stouffer RL. Differential effects of estrogen and progesterone on development of primate secondary follicles in a steroid-depleted milieu *in vitro*. Hum Reprod. (2015) 30:1907–17. doi: 10.1093/humrep/dev119, PMID: 26040480 PMC4507328

[14] Thierry H van Dessel T Schipper I Pache TD van Geldorp H de Jong FH. Normal human follicle development: an evaluation of correlations with Oestradiol, androstenedione and progesterone levels in individual follicles. Clin Endocrinol. (1996) 44:191–8. doi: 10.1046/j.1365-2265.1996.662483.x, PMID: 8849574

[15] Statello L Guo CJ Chen LL Huarte M. Gene regulation by long non-coding RNAs and its biological functions. Nat Rev Mol Cell Biol. (2021) 22:96–118. doi: 10.1038/s41580-020-00315-9, PMID: 33353982 PMC7754182

[16] Yao XL El-Samahy MA Li XD Bao YJ Guo JH Yang F. LncRNA-412.25 activates the LIF/STAT 3 signaling pathway in ovarian granulosa cells of Hu sheep by sponging miR-346. FASEB J. (2022) 36:e22467. doi: 10.1096/fj.202200632R 35929417

[17] Wang Y Guo YX Duan CH Yang RC Zhang LC Liu YQ. Long non-coding RNA GDAR regulates ovine granulosa cells apoptosis by affecting the expression of apoptosis-related genes. Int J Mol Sci. (2022) 23:20. doi: 10.3390/ijms23095183, PMID: 35563579 PMC9104640

[18] Li N Zhou YQ Cai JL Wang YF Zhou XF Hu MT. A novel trans-acting lncRNA of ACTG1 that induces the remodeling of ovarian follicles. Int J Biol Macromol. (2023) 242:125170. doi: 10.1016/j.ijbiomac.2023.125170 37276900

[19] Zhou M Liu XQ Qiukai E Shang YX Zhang XQ Liu ST. Long non-coding RNA Xist regulates oocyte loss via suppressing miR-23b-3p/miR-29a-3p maturation and upregulating STX17 in perinatal mouse ovaries. Cell Death Dis. (2021) 12:14. doi: 10.1038/s41419-021-03831-4, PMID: 34035229 PMC8149765

[20] Wang Y Guo YX Duan CH Li JJ Ji SK Yan HH. LncGSAR controls ovarian granulosa cell steroidogenesis via sponging miR-125b to activate SCAP/SREBP pathway. Int J Mol Sci. (2022) 23:20. doi: 10.3390/ijms232012132, PMID: 36293007 PMC9603659

[21] Wang F Chen XM Sun B Ma YJ Niu WB Zhai J. Hypermethylation-mediated downregulation of lncRNA PVT1 promotes granulosa cell apoptosis in premature ovarian insufficiency via interacting with Foxo3a. J Cell Physiol. (2021) 236:5162–75. doi: 10.1002/jcp.30222 33393111

[22] Huang KJ Geng JS Wang J. Long non-coding RNA RP11-552m11.4 promotes cells proliferation, migration and invasion by targeting Brca2 in ovarian Cancer. Cancer Sci. (2018) 109:1428–46. doi: 10.1111/cas.13552 29478268 PMC5980309

[23] Huang WL Zhang XX Li A Xie LL Miao XY. Differential regulation of MRNAs and lncRNAs related to lipid metabolism in Duolang and small tail Han sheep. Sci Rep. (2022) 12:12. doi: 10.1038/s41598-022-15318-z, PMID: 35778462 PMC9249921

[24] La YF Tang JS He XY Di R Wang XY Liu QY. Identification and characterization of mRNAs and lncRNAs in the uterus of Polytocous and Monotocous small tail Han sheep (*Ovis Aries*). PeerJ. (2019) 7:e6938. doi: 10.7717/peerj.6938, PMID: 31198626 PMC6535221

[25] Wang JL Chen HY Zeng XC. Identification of hub genes associated with follicle development in multiple births sheep by WGCNA. Front Vet Sci. (2022) 9:18. doi: 10.3389/fvets.2022.1057282, PMID: 36601328 PMC9806849

[26] Yao XL Yang F El-Samahy MA Liu B Zhao BR Gao XX. Identification and characterization of unique and common lncRNAs and mRNAs in the pituitary, ovary, and uterus of Hu sheep with different prolificacy. Genomics. (2022) 114:110511. doi: 10.1016/j.ygeno.2022.110511 36283658

[27] Chen X Chen HY Jiang S Shen H Zeng XC. Identification of lncRNA expression in the estrous cycle of Qira black sheep and its combination with Mirna analysis. Kafkas Univ Vet Fak Derg. (2021) 27:733–40. doi: 10.9775/kvfd.2021.26203

[28] Yan H Zhang JW Wen J Wang YB Niu WB Teng Z. CDC42 controls the activation of primordial follicles by regulating PI3k signaling in mouse oocytes. BMC Biol. (2018) 16:16. doi: 10.1186/s12915-018-0541-4, PMID: 29976179 PMC6033292

[29] Wang P Li WT Liu ZY He XY Hong QH Lan R. Identification of Wnt4 alternative splicing patterns and effects on proliferation of granulosa cells in goat. Int J Biol Macromol. (2022) 223:1230–42. doi: 10.1016/j.ijbiomac.2022.11.083, PMID: 36395931

[30] Bridges MC Daulagala AC Kourtidis A. LNCcation: lncRNA localization and function. J Cell Biol. (2021) 220:17. doi: 10.1083/jcb.202009045, PMID: 33464299 PMC7816648

[31] Li D Wang XY Li GY Dang YJ Zhao SD Qin YY. lncRNA ZNF674-AS1 regulates granulosa cell glycolysis and proliferation by interacting with ALDOA. Cell Death Discov. (2021) 7:107. doi: 10.1038/s41420-021-00493-1, PMID: 33993190 PMC8124069

[32] Yao GD Kong Y Yang G Kong DQ Xu YJ He JH. Lnc-Gulp1-2:1 affects granulosa cell proliferation by regulating Col3a1 expression and localization. J Ovarian Res. (2021) 14:10. doi: 10.1186/s13048-021-00769-1, PMID: 33472700 PMC7816396

[33] Gao M Liu LY Li SL Zhang XD Chang ZW Zhang MZ. Inhibition of cell proliferation and metastasis of human hepatocellular carcinoma by miR-137 is regulated by CDC42. Oncol Rep. (2015) 34:2523–32. doi: 10.3892/or.2015.4261, PMID: 26352279

[34] Chen HL Yang YF Wang YL Li Y He YM Duan JX. Phospholipase C inhibits apoptosis of porcine primary granulosa cells cultured *in vitro*. J Ovarian Res. (2019) 12:10. doi: 10.1186/s13048-019-0567-4, PMID: 31554511 PMC6761717

[35] Luo N Guo J Chen L Yang W Qu X Cheng Z. Arhgap10, downregulated in ovarian Cancer, suppresses Tumorigenicity of ovarian Cancer cells. Cell Death Dis. (2016) 7:10. doi: 10.1038/cddis.2015.401, PMID: 27010858 PMC4823924

[36] Zhang JQ Ma RJ Li L Wang LN Hou XJ Han LS. Intersectin 2 controls actin cap formation and meiotic division in mouse oocytes through the Cdc42 pathway. FASEB J. (2017) 31:4277–85. doi: 10.1096/fj.201700179R, PMID: 28626024

[37] Zhang JW Yan L Wang YB Zhang S Xu XQ Dai YL. *In vivo* and *in vitro* activation of dormant primordial follicles by EGF treatment in mouse and human. Clin Transl Med. (2020) 10:e182. doi: 10.1002/ctm2.182, PMID: 32997412 PMC7520080

[38] Tian YQ Li XL Wang WJ Hao HS Zou HY Pang YW. Knockdown of bone morphogenetic protein 4 gene induces apoptosis and inhibits proliferation of bovine cumulus cells. Theriogenology. (2022) 188:28–36. doi: 10.1016/j.theriogenology.2022.05.015, PMID: 35661480

[39] Xu RF Qin N Xu XX Sun X Chen XX Zhao JH. Inhibitory effect of SLIT2 on granulosa cell proliferation mediated by the CDC42-PAKs-ERK1/2 MAPK pathway in the Prehierarchical follicles of the chicken ovary. Sci Rep. (2018) 8:16. doi: 10.1038/s41598-018-27601-z, PMID: 29907785 PMC6003946

[40] Rivera HM Bethea CL. Ovarian steroids increase Spinogenetic proteins in the macaque dorsal raphe. Neuroscience. (2012) 208:27–40. doi: 10.1016/j.neuroscience.2012.02.002, PMID: 22342969 PMC3312750

[41] Sukocheva O Wadham C Xia P. Estrogen defines the dynamics and destination of Transactivated EGF receptor in breast Cancer cells: role of S1P3 receptor and CDC42. Exp Cell Res. (2013) 319:455–65. doi: 10.1016/j.yexcr.2012.10.014, PMID: 23142484

